# Efferent control of hair cells mechanically coupled by artificial membranes

**DOI:** 10.1038/s41598-025-31059-1

**Published:** 2025-12-13

**Authors:** Martín A. Toderi, Gabriela Muñoz-Hernandez, Justin Faber, Dolores Bozovic

**Affiliations:** 1 Department of Physics & Astronomy, University of California, Los Angeles, Los Angeles, CA 90095 USA; 2 California NanoSystems Institute, University of California, Los Angeles, CA 90095 USA

**Keywords:** Sensory systems, Hair cells, Efferent feedback, Coupled hair bundles, Biological physics, Hair cell, Transduction, Sensory processing

## Abstract

The efferent system has been proposed to play a vital function in auditory and vestibular systems, by protecting the sensory hair cells from injury and preserving signal detection sensitivity. We report that the activation of efferents has strong modulatory effects on systems of coupled hair cell bundles. In this study, we use direct electrical stimulation of efferent neurons to probe its effects on the hair cells’ internal dynamics, by means of optically tracking the hair bundle motility. *In vivo*, hair bundles of auditory and vestibular epithelia are connected via overlying membranes; to address this physiological characteristic, we investigate the impact of efferent activity on the collective response of coupled hair bundles. We use a preparation from the American bullfrog sacculus which preserves the active motility of hair bundles, and achieve inter-cell coupling by connecting the cells to artificial mica structures. We found that efferent stimulation impacts hair-bundle dynamics, affecting the amplitude, frequency, and temporal profile of spontaneous oscillations, and altering the dynamical state of the system. Furthermore, efferent activation decreased synchronization among coupled hair bundles, suggesting a mechanism by which neural modulation may reduce the overall sensitivity of the system.

## Introduction

**Fig. 1:**
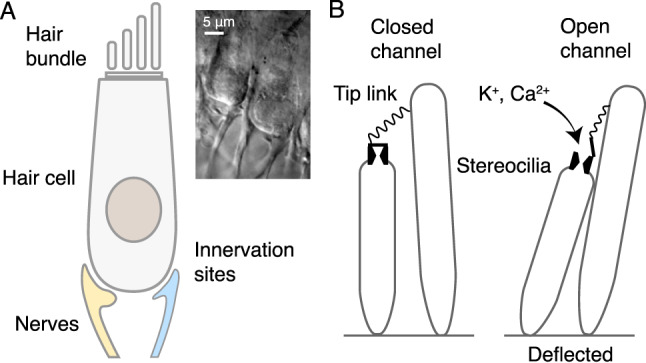
(**A**) Schematic diagram of a frog’s vestibular Type II hair cell and the innervating neurons. The cluster of stereocilia at the apical surface comprise the mechanosensitive organelle of a hair cell, the hair bundle. Synaptic contacts are present at the basal pole of the hair cell soma. The inset is a DIC image of the side view of a hair cell from the bullfrog’s amphibian papilla together with the nerve fibers. (**B**) Schematic diagram of the transduction process. Stereocilia are inter-connected by tip links, which are mechanically coupled to transduction channels. Upon increase in the tension of the tip link due to deflection, the transduction channel opens, allowing the influx of ions.

A vast majority of living organisms within the animal kingdom rely on their auditory and vestibular systems to communicate, navigate, and avoid danger. These characteristics depend on the capacity to extract information from surroundings with several competing streams of information, as well as having enough sensitivity to detect extremely weak signals^1^. Vertebrates’ ability to hear and balance is rendered possible through specialized cells present in internal sensory organs within the inner ear^2^. Hair cells are the key functional units that execute the initial stage of detection. Protruding from the apical surface of the hair cell is a bundle of stereocilia, which is collectively named the hair bundle (Fig. 1A).

Hair cells in the vestibular and auditory systems are extraordinary sensory organs, capable of detecting mechanical displacements of the hair bundle with subnanometer sensitivity. Deflection of the stereocilia opens mechano-sensitive transduction channels (Fig. 1B), eliciting spike trains in the innervating afferent neurons^3,4^. The opening probabilities of the transduction channels are regulated by the tension in the tip links connecting adjacent stereocilia. This tension is in turn influenced by myosin motors that climb and slip along the actin filaments that comprise the core of the stereocilia, allowing the hair bundle to adapt to continuous deflection. The system incorporates an active amplifier, which uses energy to improve the responsiveness to extremely small signals. In certain species, an active process has been shown to result in spontaneous oscillations of the hair bundle^5^.

In hair bundles of the bullfrog’s sacculus, prior studies have shown that the mechanical gating of the transduction channels leads to a regime of effective negative stiffness. When channels open in response to a stimulus, the corresponding gating swing softens the tension in the tip link, allowing the bundle to move further along the stimulus direction. The adaptation motors then readjust the tension, allowing the channels to reclose^6^. Each cycle of oscillation represents a trajectory around a displacement–response relation that determines the characteristic motion trace of the hair bundles. Specifically, the bullfrog sacculus is sensitive to seismic vibrations and airborne sounds in the 5–200 Hz frequency range, with free-standing hair bundles exhibiting spontaneous oscillations between 5 and 100 Hz^5,7^. These limit cycle oscillations can be altered by mechanical, electrical, and chemical manipulations and provide an experimental probe of the hair bundle’s internal dynamics.

### Systems of coupled hair bundles

Different species exhibit a broad variation in the extent of inter-cell coupling within their inner-ear organs^8^. While hair bundles of certain end organs are free-standing^9^, others are connected by overlying structures, either in small clusters by sallets, or in larger numbers, through membranes that cover most or all of the sensory epithelium^10,11^. Differences in the thickness and shape of the overlying membranes result in varying levels of coupling strength. In the bullfrog sacculus, the otolithic membrane imposes strong coupling on the hair bundles, while allowing deflections along the axis of sensitivity^12^. Experiments performed on the bullfrog sacculus probed for signatures of the active process under natural loading and coupling conditions. Transient mechanical stimuli applied to the otolithic membrane were shown to elicit active twitches analogous to those observed in individual hair bundles^13^. Furthermore, in the presence of loading, the coupled bundles showed a decreased frequency selectivity compared to that of free-standing cells. The tectorial membrane in mammals varies in its characteristics, with a decreasing transverse thickness and increasing width from the base to the apex of the cochlea^14^. The different types and degrees of connection in various end organs prompted inquiries into how this connection impacts the active motion of hair bundles.

Theoretical investigations on networks of interconnected nonlinear oscillators, each located close to a Hopf bifurcation, showed that noise removed the key signatures of criticality in the dynamics of each oscillator^15^. However, an array of numerous oscillators exhibiting interaction with their closest neighbors leads to synchronization across significant distances and restores key signatures of criticality. Results from experiments performed on the mammalian cochlea show a one-third power-law relationship across several orders of magnitude of stimulus amplitude^16^, consistent with the proposed proximity to the critical point^17^. A different study investigated a more intricate biophysical model of hair bundle mechanics and introduced elastic springs as connecting elements between closest neighbors^18^. The coupling increases the sensitivity of the system, as well as the range and extent of nonlinearity. Theoretical studies have therefore shown that coupling observed in sensory end organs plays an important role in shaping the dynamics of the full system.

Experimental studies have also explored the role of inter-cell coupling in a system in which a biological hair bundle was monitored in real-time and digitally connected to a numerical simulation mimicking its dynamics through a feedback system^19^. The spontaneous oscillations of the real and virtual cell became synchronized as their coupling increased, resulting in an increase in the sensitivity, quality factor, and the range of compressive nonlinearity, corroborating the theoretical predictions.

### Efferent activity

The efferent neurons act as an otoprotective feedback system, sending information from the brain’s higher processing areas to the sensory epithelium^20,21^. Earlier research has shown that efferents modulate the sensitivity of the auditory system’s detection^22^ and protect it from damage caused by moderate and loud noise^23^. Experimental measurements also showed that efferents can reduce basilar membrane movement elicited by incoming sound^24^. In the auditory system, the efferent neurons also play a role in aiding communication and navigational abilities^25,26^, and were shown to be involved in an animal’s ability to isolate a selected auditory stream from a complex acoustic environment^27^. Nevertheless, despite the ample experimental proof of the significance of efference for the auditory sense, there are still many unknown aspects of how it exerts its influence. Electrophysiological recordings in the bullfrog sacculus on the hair cell soma showed that most of the hair cells experienced hyperpolarization due to efferent activation^28^.

Previous research has demonstrated that individual hair bundles consistently exhibit a decreased mechanical sensitivity upon efferent stimulation^21,29^. We proposed a theoretical framework describing how efferent input impacts the dynamics of an active hair bundle^30^, which further demonstrated its importance. In this manuscript, we explore the effect of efferent activation on systems of hair cells, mechanically coupled with artificial membranes. As hair bundles *in vivo* experience various degrees of inter-cell coupling, we aim to experimentally probe how signals from the brain impact the collective mechanics of active hair bundles.

## Methods

We confirmed that all experiments in this study were performed in accordance with the relevant guidelines and regulations. All the procedures of the study followed the ARRIVE guidelines.

### Sample preparation

**Fig. 2:**
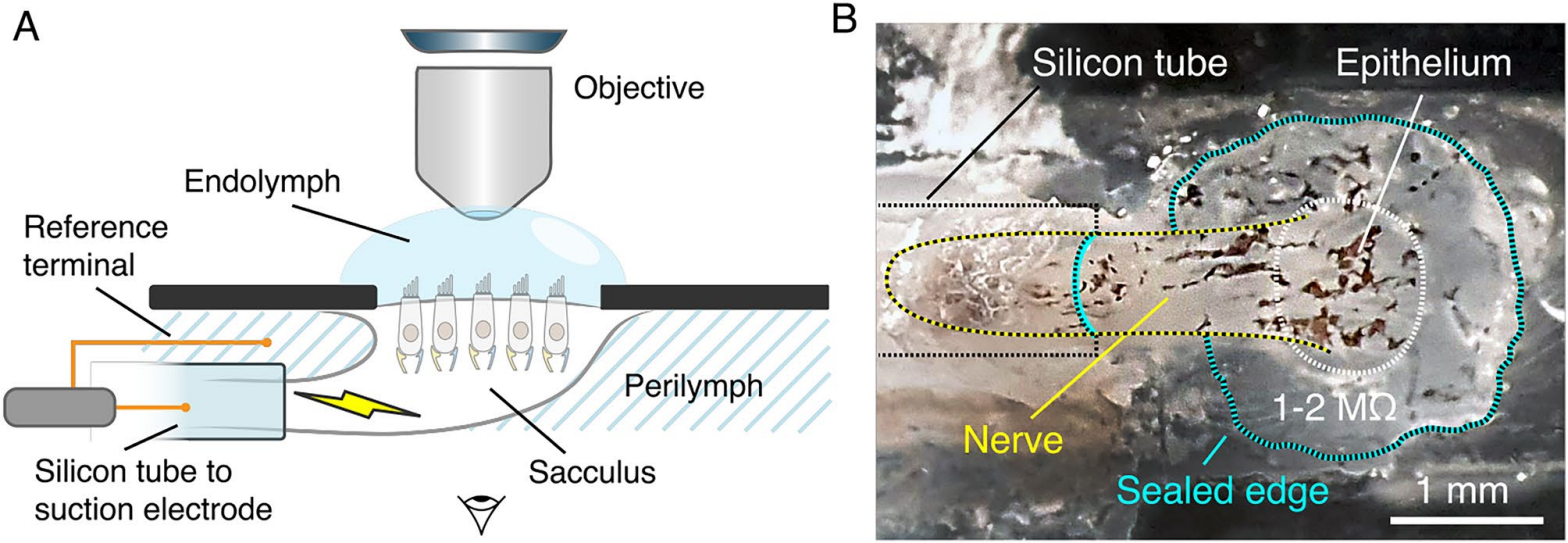
(**A**) Two-compartment chamber emulating the ionic concentrations of the natural fluid environment of the sacculus. The nerves are immersed in perilymph at the bottom compartment, and the hair bundles in endolymph at the top. (**B**) Bottom view of the preparation, the eighth cranial nerve is left long enough to be inserted into a silicon tube, the perilymph that fills the tube is in contact with the positive terminal of the electrode and the nerve, and isolated from the bath with a glue seal.

Hair cells from dissected sacculi of the North American Bullfrog (Frog Pharm, Twin Falls ID, USA) were imaged *ex vivo* while maintaining their physiological integrity. The study was approved by the Chancellor’s Animals Research Committee at the University of California, Los Angeles (Protocol number: ARC-2006-043-TR-001). All procedures were conducted in accordance with the ethical standards and guidelines authorized by the Chancellor’s Animals Research Committee at the University of California, Los Angeles. Frogs were anesthetized (using 150 mg/kg of pentobarbital), double-pithed, and beheaded. Sacculi were removed from the animals’ inner ears and put in an oxygenated artificial perilymph solution (in mM as follows: 110 Na$^{+}$, 2 K$^{+}$, 1.5 Ca$^{2+}$, 113 Cl$^{-}$, 3 D-(${+}$)-glucose, 1 Na$^{+}$ pyruvate, 1 creatine, 5 HEPES). In order to replicate the fluid partitioning of the physiological conditions found *in vivo*, the epithelium was placed in a chamber with two compartments. Apical areas were covered in artificial endolymph (in mM as follows: 2 Na$^{+}$, 118 K$^{+}$, 0.25 Ca$^{2+}$, 118 Cl$^{-}$, 3 D-(${+}$)-glucose, 5 HEPES) and basolateral membranes in perilymph, as depicted in Fig. 2A. The otolithic membrane was carefully separated from the epithelium following an 8-minute enzymatic dissociation with 15 g/ml Collagenase IV (Sigma-Aldrich) to enable direct mechanical access to the hair bundles. Upon membrane removal, free-standing hair bundles exhibit spontaneous oscillations.

### Efferent stimulation and artificial mechanical coupling

A bipolar suction electrode (A-M Systems) was used to introduce external electrical stimuli. The eighth cranial nerve was inserted into a 0.51-mm-internal-diameter silicon tube (HelixMark) (Fig. 2B), which was electrically connected to the positive terminal of the electrode, while the reference terminal was immersed in the perilymph compartment of the chamber (Fig. 2A)^21^. All of the nerve’s efferent neurons were activated simultaneously as a result of this method. A linear stimulus isolator (World Precision Instruments A395) provided current to the suction electrode, and stimulus protocols were sent to the isolator via LabView (National Instruments cDAQ-9178). The resistances of the preparations were measured in the 1–2 M$\Omega$ range. Immediately after the efferent stimulus began, altered hair bundle oscillations were observed and ceased upon the electric signal termination. Stimulus was sent in the form of square pulse trains of 5, 20, 40, 60, 80, and 100 Hz at 50% duty cycle of 100 µA intensity, and as continuous steps of 25, 50, 75, 100, 150 and 200 µA during 10 s. To observe the changes introduced by efferent activation, periods of no stimulation were left before and after the current was applied, also of 10 s each. In a prior study, it was shown that this method does not elicit a spurious effect, which would directly electrically stimulate the hair-cell bundle or soma^21,29^. After introducing strychnine, a known blocker of acetylcholine receptors, into the surrounding fluid, hair-bundle oscillations persisted, while the external electrical stimulus yielded no effect on the hair-bundle dynamics. This control was fully reversible, as the effects of the electrical stimulus could be recovered upon washing out the strychnine. The same procedure was followed for the coupled system setup, as detailed in Section 2 of the Supplementary Information, and shown in Fig. S2.

*In vivo*, the otolithic membrane links hair bundles through filaments attached to the tips of the stereocilia, with variations in the thickness of the membrane forming fluid-filled “pits” above the hair bundles. The structure of this overlaying membrane introduces mechanical loading that tunes the hair bundles into the quiescent regime^13^. Our preparation introduces coupling between cells by connecting free-standing stereocilia to overlying mica by direct contact. The mechanical load is selected to be sufficiently strong to introduce inter-cell coupling but not to suppress the spontaneous oscillatory regime. This hybrid preparation thus allows us to analyze the synchronization of oscillations and to control the size of the coupled array. Hence, while it does not aim to fully reproduce specific loading conditions of the sacculus *in vivo*, it aims to explore the general impact of coupling on the dynamics of hair cells. We found that varying the system size, over the range of 3-13 cells, did not produce significant trends on the calculated indices, throughout the experiments with different current intensities (see Fig. S1). We deemed correlation, together with frequency and amplitude of oscillations, to be independent of the number of hair bundles in the coupled system. This allowed us to pool the results obtained from various sizes of the coupled systems and compare the differences between just two groups, coupled and controls.

A solution of mica powder (Black Diamond Pigments) and artificial endolymph was filtered through multiple stainless steel mesh sheets (74 and 100 µm sizes from Utah Biodiesel Supply) with the use of a vacuum, while checking pH stability. The filtering process extracts the desired mica flake sizes, which ranged between 20 and 80 µm, making them the ideal sizes for constraining our system to about 5-20 hair bundles lying underneath the artificial membrane with a few outlying control cells remaining within the imaging frame. The artificial membrane solution was oxygenated and then pipetted onto the biological preparation. Mica flakes were scattered at random across the cells and, when they settled, adhered to the hair bundles underneath. We recorded the coupled hair bundles’ active motility by optically tracking them through the transparent membranes. In order to provide free-standing non-coupled and coupled cells for direct comparison, hair cells that were not under the mica were included within the same field of view receiving the same efferent stimulus.

### Data acquisition and processing

**Fig. 3:**
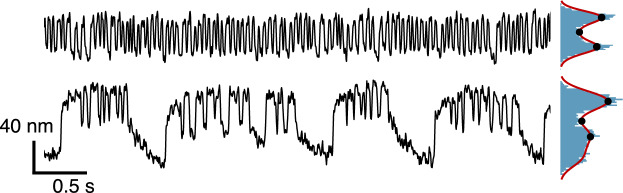
Hair bundle position traces for two different free oscillating cells; bi-modality is observed in the position histogram on the right. After a kernel density estimation fit (red), reference points for amplitude and open probability calculations are marked in black. The first trace yields a dip statistic of 0.0474 and the second trace 0.0232, classifying both of them as oscillatory.

Experiments were performed on an upright optical microscope (Olympus BX51WI) with a water-immersion objective (Olympus LUMPlanFL N 60X, NA: 1.00) and standard differential-interference-contrast optics, mounted on an optical table (Technical Manufacturing). To prevent outside disturbances to the extremely sensitive hair cells, the setup was built inside an acoustically isolated chamber (Industrial Acoustics). Images at a resolution of 108.3 nm/px were recorded with a high speed camera (ORCA-Flash4.0 CMOS) at 400 to 1000 frames per second. For each set of experiments, motion of the hair bundles was tracked according to previously developed image processing methods using custom made MATLAB scripts^31^. For each frame, centroid weights are set proportional to pixel intensity, and the position is found by a 2D Gaussian fit employing the Levenberg-Marquardt algorithm. Our method finds the most prominent direction of motion and determines the motion trace for each tracked hair bundle. Motion traces are post-processed to eliminate possible drift due to gradual relaxation of the preparation over the course of the recording. This removes undesired low-frequency fluctuations of the trace by a polynomial fit or moving average applied in series, obtaining stationary signals for each experimental record. Figure 3 shows two typical spontaneous oscillatory regimes obtained from unperturbed free-standing hair bundles. For the coupled system experiments, hair bundle motion traces were then split into three sections, aligned with recordings taken before, during, and after the application of efferent stimulus, and each section was analyzed separately. In line with previous studies, movement towards the kinocilium, shown to correspond to the opening of transduction channels, is associated with positive displacement in the traces. Data were obtained from 191 hair bundles recorded across six areas per four different epithelia.

#### Parameters calculation

**Fig. 4:**
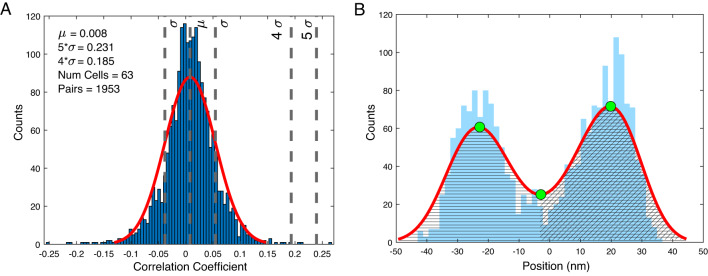
(**A**) Correlation coefficient distribution obtained from a selected recording of 63 hair bundles. The motion traces are compared to each other, yielding 1953 pairs. A nonparametric kernel-smoothing distribution (red line) fits the histogram for estimation of the standard deviation $\sigma$. Four different sacculi are used to calculate this threshold for coupling, which is established at 0.2, falling between 4 and 5 $\sigma$. (**B**) Position distribution for a trace exhibiting bimodality. The red line is the kernel-smoothing fit and the green dots mark the maxima and minimum established to calculate the amplitude and the open probability approximation. The open probability is calculated as the quotient between the integral of the positive position section (cross shaded) and the full distribution (horizontally shaded).

Due to the differences in heights of the stereocilia, not every hair bundle within a group allowed full contact with the artificial membrane above it. The degree of connection among cells was evaluated by calculating the cross correlation between individual traces as well as with the artificial membrane. The correlation coefficient threshold to determine coupling is 0.2, calculated from 4 experiments for uncoupled saccular cells (distribution of $\approx$ 2000 pairs each). We recorded free spontaneous oscillations of hair bundles and calculated all the normalized cross-correlation coefficients for possible pairs as,

1 $$\begin{aligned} cc (x,y)= \frac{cov(x,y)}{\sigma _{x}\sigma _{y}}, \end{aligned}$$

where *cov*(*x*, *y*) denotes the covariance between traces x and y, and $\sigma$ is the standard deviation. This yielded a distribution of correlation coefficients, an example of which is shown in Fig. 4A. Fitting the histogram yields a standard deviation value of approximately $\sigma$ = 0.046, from which we established the coupling threshold, between 4$\sigma$ and 5$\sigma$, at 0.2. For this threshold, the probability that uncoupled oscillating cells exhibit spurious correlation of their motion higher than 0.2 is 0.0063%. Given this threshold, we then assume that a higher correlation observed between bundles underneath a mica flake to indicate a mechanical link between pairs of hair bundles.

Position traces of coupled and control hair bundles were analyzed so as to characterize how efferent activation influences the cells’ spontaneous oscillation. A kernel smoothing function is applied to the position distribution of the hair bundle motion (Fig. 4B). Traces are classified as oscillatory if the Hartigans’ dip test^32^ exceeds 0.01 with a corresponding p-value under 0.001, and if the algorithm can find two local maxima and one minimum to define the amplitude. The amplitude was set to be half of the distance between the two local maxima in the position distribution. An estimate of the transduction channels open probability was extracted from the position traces of the hair bundles. The average open probability was calculated by determining the region under the positive position area, divided by the region under the full probability density function. The frequency was defined as the average of the instant frequency obtained from the Hilbert Transform, calculated as the derivative of the phase of the analytic signal. All parameters are presented with their standard deviation from the mean.

*Kolmogorov entropy*

The Kolmogorov entropy, metric entropy, or Kolmogorov-Sinai entropy is an information-theoretical measure of chaos in dynamical systems^33,34^. Its value is zero for non-chaotic systems, positive for chaotic systems, and diverges toward infinity for stochastic white noise. Chaotic dynamical systems exhibit sensitivity to initial conditions, resulting in the divergence of neighboring trajectories. With finite measurement precision, neighboring points in phase space may be indistinguishable and regarded as identical. In this sense, the Kolmogorov entropy can be thought of as the rate at which the system is producing new information, as a result of the uncertainty expanding with time.

To calculate the Kolmogorov entropy from time series data, we must first reconstruct the phase space of the dynamical system using embedding techniques^35,36^. We follow previously described procedures^37^, using the first zero-crossing in the autocorrelation function as the time delay, $\tau$, and $D=5$ embedding dimensions. Note that the results do not qualitatively change upon adjusting these parameters. We then partition the phase space into hypercubes of uniform volume, $\epsilon ^D$. Due to the bimodal distribution of hair-bundle position, we choose just two partitions along each dimension of the reconstructed phase space. This results in a total of $2^D = 32$ phase-space hypercubes, which the dynamical system may occupy.

At a given instance in time, we can calculate the Shannon entropy associated with the distribution of trajectories through phase space,

2 $$\begin{aligned} S_n = -\sum _{i_0,..., i_n} P_{i_0,..., i_n}\log P_{i_0,..., i_n} \end{aligned}$$

where $P_{i_0,..., i_n}$ is the probability of finding the system in state $i_0$ at $t=0$, $i_1$ at $t=\tau$, $i_2$ at $t=2\tau$, and so on. The Kolmogorov entropy is then defined as

3 $$\begin{aligned} K = \lim _{\epsilon \rightarrow 0} \lim _{N \rightarrow \infty } \frac{1}{N\tau } \sum _{n=0}^{N-1} (S_{n+1} - S_n). \end{aligned}$$

In practice, we must take partitions larger than the noise floor, and truncate the sum before trajectories have fully diverged. For this reason, we consider only the initial divergence of the trajectories, $K \approx \frac{1}{\tau }(S_1 - S_0)$. Further, we report the measure in time units, $\tau$, that are intrinsic to each particular hair bundle. Thus, a simple change in a hair bundle’s characteristic frequency does not influence our measure of Kolmogorov entropy.

## Results

The bullfrog’s saccular hair cells receive dense efferent innervation^28^. Efferents trigger the release of acetylcholine (ACh). Thereupon, ACh binds to nicotinic receptors in the hair cell soma, triggering the influx of Ca$^{2+}$, which activates SK2 K$^{+}$ channels. The subsequent efflux of K$^{+}$ causes hyperpolarization of the membrane potential^38^. The induced changes in the somatic potential affect the hair bundle motility, altering the patterns of spontaneous oscillations^21^. Our *ex vivo* experimental arrangement allows for electrical activation of efferents, simulating the neural feedback control of hair cell dynamics. Electrical stimulus is applied within the perilymph compartment, thus constraining the currents to the fluid bathing the cell somae and innervating neurons. Changes in spontaneous oscillations induced by efferent activation are consistent with hyperpolarization of the membrane potential via patch-clamp^39^ or those elicited by transepithelial currents^40^. We probed the hair cells’ internal dynamics in the coupled system by analyzing the alterations of these spontaneous oscillations, which are a direct consequence of the instability induced by their active nature. Efferent stimulation was found to affect the active motion of the hair bundles, changing the level of synchronization, frequency pattern, and oscillation amplitude, with specific changes dependent on the form of the stimulus and coupling.

### Pulse train efferent stimulation

**Fig. 5:**
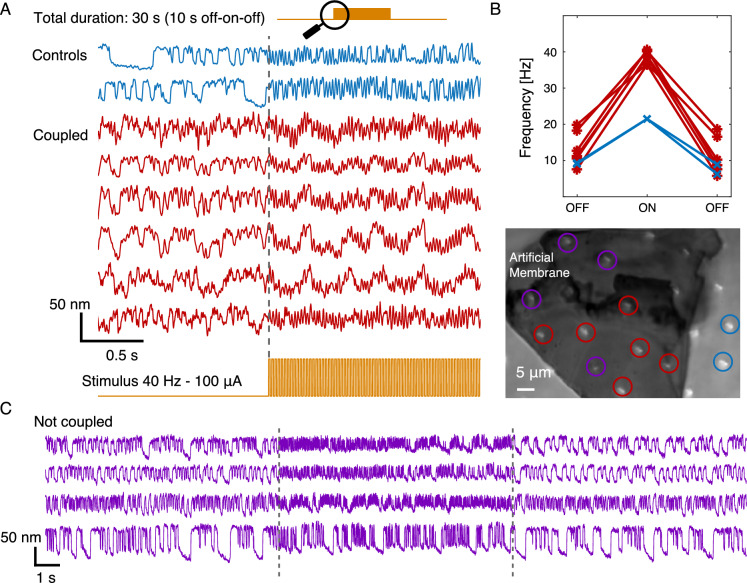
(**A**) Example traces of spontaneous oscillation, obtained during efferent modulation (pulse train: 40 Hz, 100 µA), are displayed for six hair bundles imaged through the artificial membrane (red) and two control cells outside the membrane area (blue). Atop there is a schematics of the portion of the experiment shown. The shown portion zooms into the transition area at the onset of the stimulus for viable observation of oscillations. The bottom trace illustrates the shape of the stimulus. The traces have arbitrary vertical offsets for clarity. The efferent stimulus commenced at the gray vertical dashed line mark. (**B**) Red (*) shows the characteristic frequency for hair bundles coupled to the membrane before, during and after the stimulus, while blue (x) marks the control. During efferent activation, the frequency of the hair bundles under the artificial membrane converges to the stimulus frequency, in this case 40 Hz; the control hair bundle increases in frequency but does not phase-lock to the stimulus. The bottom panel shows an image (60X objective) of the artificial membrane and the cells with color coded circles labeling each cell (coupled, not coupled and control). (**C**) Spontaneous oscillations of cells that were not classified as coupled but are under the membrane (purple). The dashed lines mark the efferent stimulus (40 Hz–100 µA) interval.

Hair bundles under the artificial membrane showed robust phase-locking to the electrical square-wave pulse trains applied to the efferent neurons. Stimulus signals were sent with frequencies of 5, 20, 40, 60, 80, and 100 Hz, at 100 µA intensity. The correlation coefficient (CC) quantified an increase in the synchronization of motion between coupled cells and the artificial membrane when efferents were activated. Figure 5 shows traces of selected bundle oscillations, recorded while efferents were stimulated with a 40 Hz pulse train. Interestingly, phase-locking was not consistently observed for control cells, with a notable disparity at the higher end of the stimulus frequencies, throughout the pulse train experiments. Coupling between hair bundles therefore facilitated entrainment to efferent signals at higher frequencies of stimulation. The hair cells returned to their original state upon termination of the stimulus. Additionally, Fig. 5C shows traces of cells that lie beneath the artificial membrane, but are classified as not coupled based on the CC values.

**Fig. 6:**
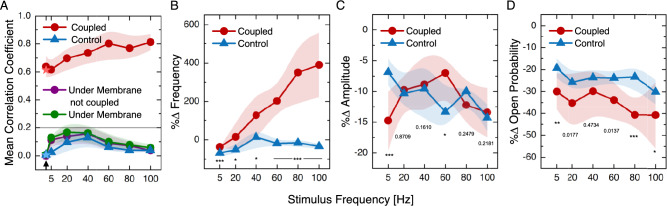
Changes of bundle motion parameters with increasing stimulus frequency. The efferent pulse trains were applied at an intensity of 100 µA, 50% duty cycle, square shape, and their frequency varied from 5 to 100 Hz. 113 traces were analyzed from four areas per three different epithelia. Dispersion of values (Standard Deviation) is depicted by light colored envelopes. (**A**) shows the CC for coupled cells (red) i.e., pairs of cells exhibiting a coefficient higher than 0.2 with respect of the artificial membrane motion. The first x-axis tick with the arrow denotes the non-stimulated case, the gray line marks the threshold. There is an increasing trend as the frequency of the stimulus grows. The CC for all bundles under the artificial membrane, uncoupled bundles under the artificial membrane and control bundles are shown in green, purple and blue respectively. As expected, these groups stay within the non-coupled regime, below 0.2. Nevertheless, one can note a bump in the CC for frequencies close to the typical spontaneous oscillation frequencies of the sacculus (10–30 Hz). $\star$ marks the final state after terminating stimulation. (**B**–**D**) show the parameters percentage change, exhibiting an increase or decrease at the onset of efferent stimulation with respect to the non-stimulated state. Two-sample t-test p values are annotated for each stimulus frequency, p < 0.05 (*), p < 0.01 (**), p < 0.001 (***). Coupled cells are in (red) and control cells in (blue).

Figure 6 summarizes the impact of increasing the frequency of the efferent stimulus on the parameters characterizing bundle oscillation. Coupled cells exhibited stronger effects due to efferent stimulation than the control cells, particularly increasing inter-cell synchronization (Fig. 6A, average CC) and frequency of oscillation (Fig. 6B), reflecting entrainment to the efferent signal. The purple trace in Fig. 6A corresponds to mean CCs from cells beneath the membrane that oscillate but remain below the coupling threshold. We note that, due to membrane unevenness and hair bundle height differences, a number of cells show CC below the defined threshold for coupling. As these cells’ mechanical contact is uncertain, and their responses may be influenced by partial coupling or proximity effects to the membrane, they are excluded from further analysis. Both control and coupled cells showed a decreasing trend in the percentage variations of amplitude, without any notable difference due to the coupling (Fig. 6C). Coupled cells presented greater reductions of the opening probability with efferent activation, the effect being milder in control cells (Fig. 6D).

### Continuous efferent stimulation

**Fig. 7:**
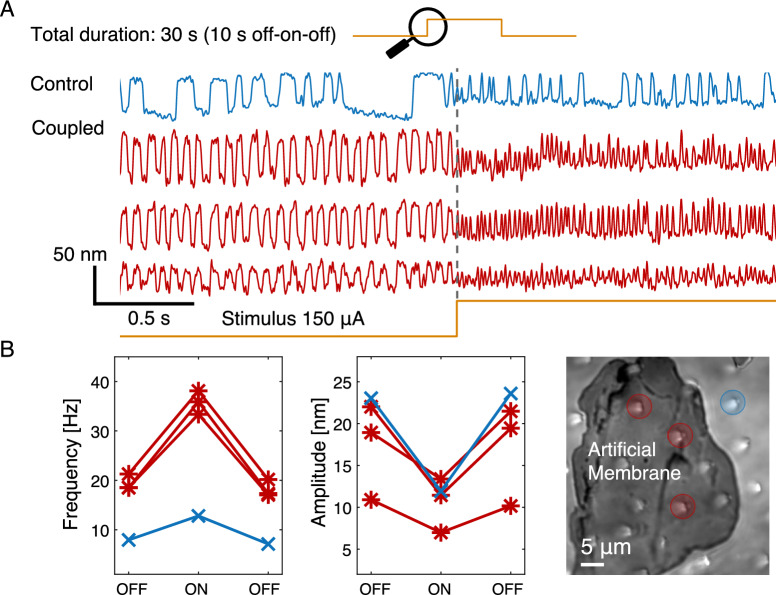
(**A**) Example traces of spontaneous oscillations, obtained before and during efferent modulation (continuous step 150 µA), are displayed for three hair bundles under the artificial membrane (red) and a control cell outside the membrane area (blue). Atop there is a schematic of the portion of the experiment shown. The shown portion zooms into the transition area at the onset of the stimulus for viable observation of oscillations. The stimulus waveform is depicted at the bottom. The traces have arbitrary vertical offsets for clarity. The efferent stimulus commenced at the gray vertical dashed line mark. (**B**) Red (*) show the characteristic frequency and amplitude for hair bundles coupled to the membrane before, during and after the stimulus, while blue (x) marks the control. The rightmost panel shows an image (60X objective) of the artificial membrane and the cells with color coded circles labeling each cell (coupled and control).

**Fig. 8:**
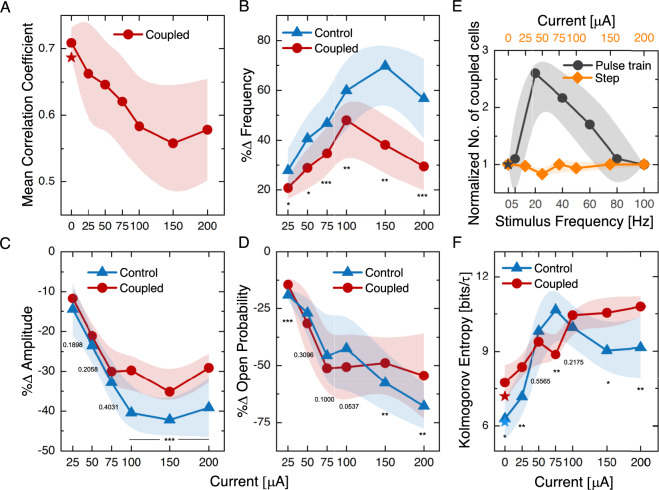
Changes in hair bundle motion parameters with step-shaped efferent stimulation. The efferent continuous step intensity spanned from 25 to 200 µA. 191 traces were analyzed from six areas per four different epithelia. Dispersion of values (Standard Deviation) is depicted by light colored envelopes. (**A**) Shows the CC for coupled cells (red). There is a decreasing trend as the intensity of the step stimulus grows, $\star$ marks the final state after terminating stimulation. (**B**–**D**) show the parameters percentage change, upon the onset of efferent stimulation, with respect to the non-stimulated state. Note that the coupled cells show a milder effect. Coupled cells are shown in (red) and control cells in (blue). Two-sample t-test p values are annotated for each stimulus current, p < 0.05 (*), p < 0.01 (**), p < 0.001 (***). (**E**) The number of coupled cells, normalized to the initial state, is plotted for both kinds of stimuli, pulse train and step. Note the different x-axis for the two sets of experiments. Pulse train experiments exhibit $\approx$ 3-fold increase in the number of cells exceeding the synchronization threshold, reflecting entrainment to the common signal, that of the efferent stimulus. The step stimulation exerts no effect on the number of coupled cells above the synchronization threshold. (**F**) Kolmogorov entropy calculated for continuous step experiments. The values increase with current intensity, with a steeper growth observed for control cells; $\star$ marks the final state after terminating stimulation.

In this set of experiments, we activated the efferent neurons in a manner that did not introduce a particular frequency component, and assessed their effect on spontaneous oscillation profiles and inter-cell synchronization. Continuous efferent activation was induced in the nerve by applying a prolonged step stimulus of 10 s duration, at 25, 50, 75, 100, 150 and 200 µA intensity. An experiment corresponding to a 150 µA step stimulus is depicted in Fig. 7. Figure 7A exhibits selected illustrative traces from the experiment indicated in Figure 7B (rightmost panel). The frequency and amplitude of these oscillations were strongly affected by efferent activation; further, coupled cells responded differently from the controls (Fig. 7B). The effects on inter-cell synchronization and overall oscillation characteristics were substantially different in this scenario (Fig. 8A–D) than those observed when the efferents were stimulated by a pulse train. In the absence of external entrainment, synchronization was due to a competitive interplay between the mechanically coupled bundles. As the motion of the membrane is dictated by the overall contributions of diverse individual oscillators, hair bundles that exhibit more prominent spontaneous motility can have a greater impact on the global oscillation. Effects of efferent activation on the coupled system were consistent with their modulation of the oscillation profiles of individual cells.

Previous studies performed on individual hair bundles have shown that efferent activity strongly impacts spontaneous oscillations^21,29^. In our measurements, coupled hair bundles exhibited the same overall trends, however with weaker induced changes that those observed in the controls. Specifically, the frequency of oscillation consistently increased for both coupled cells and controls, but with a milder effect observed in the former group (Fig. 8B). Likewise, the amplitude of motion was reduced by efferent activation, with a stronger effect evident for the control cells (Fig. 8C). As the stimulus current increased, the mean opening probability was reduced, resulting in spike-shaped oscillations, with the bundles spending less time in the open channel state. No significant difference in the effects of efferent stimulus on this parameter was observed between the two populations of cells (Fig. 8D). We observed changes in the slope of parameters versus efferent stimulus for currents higher than 100 µA. These inflection points occurred for both coupled and control bundles.

The average CC, computed over all pairs of coupled hair bundles, showed a decreasing trend with efferent activation, with a plateau at current intensities above 100 µA. This trend implies an overall reduction in the synchronicity of the hair bundles (Fig. 8A).

We next tested whether the application of efferent stimulus affects the number of synchronized hair bundles. The initial number of cells, exhibiting a CC with the artificial membrane that was higher than 0.2, was taken as the baseline. Subsequently, the number of coupled cells was represented as a ratio with respect to the initial state. This metric hence tracked any change in the ability of cells to synchronize to the artificial membrane. Figure 8E shows that this parameter was invariant for the step stimulus, indicating that efferent activation was neither creating nor destroying pairs of coupled cells.

### Efferent control of Kolmogorov entropy

As an additional metric for characterizing the dynamics of coupled hair-bundles, we measured the effects of efferent activation on the Kolmogorov entropy of the system. The Kolmogorov entropy (KE) is used to quantify the rate of information production by a dynamical system^33,34^. Periodic, limit-cycle oscillations have a KE of zero, while chaotic dynamics and stochastic processes produce a positive KE, with larger values indicating more erratic oscillations (see Methods). This metric has been shown to be associated with the sensitivity and temporal acuity of hair-cells^41^. We therefore tested the possibility that efferent activation modulates the KE of the hair-bundle dynamics, as the efferent system constitutes one of the biological feedback mechanisms proposed to modulate the sensitivity of hearing.

We applied step-like signals of constant-current to the efferent neurons and measured the variation of KE with increasing stimulus levels. As can be seen in Fig. 8F, the KE increased with increasing current intensity of the efferent activation, presenting a weaker initial growth for coupled hair bundles as compared to the controls. Above currents of 75 to 100 µA, this tendency was reversed, with coupled hair bundles more strongly affected, and reaching a plateau at higher currents. The effect was fully reversible, with the system returning to the initial KE values upon cessation of the stimulus. For sufficiently high currents, efferent pulse trains result in hair bundle entrainment, and thus yield a KE of zero (not shown). We therefore omit this stimulus type from our analysis of the degree of chaos in this system.

### Partial synchronization & efferent control

**Fig. 9:**
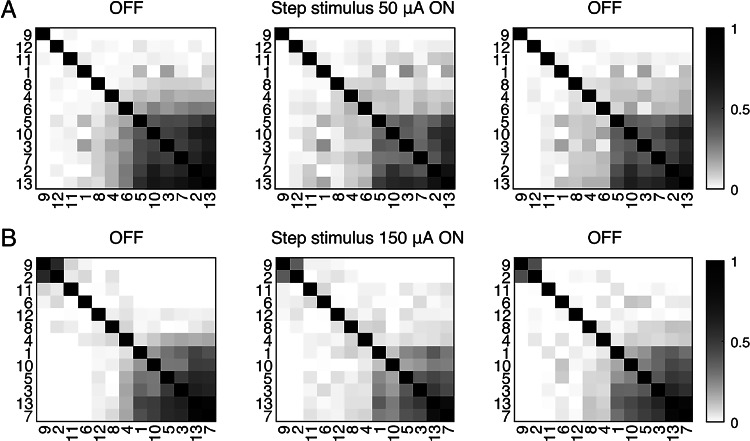
Correlation matrices of hair-bundle oscillations, with darker shades of gray denoting more strongly correlated pairs. The CCs are rearranged to group higher values together by averaging the rows in the original scrambled matrix. The numbers uniquely identify each hair bundle recorded under the artificial membrane. (**A**) Invariant configuration of synchronization with a slight decrease of the average CC for a 50 µA step stimulus. (**B**) Chimera states show no variation upon a 150 µA step stimulation. A rarely observed small secondary cluster can be seen in the upper left corner.

Partial synchronization in the form of chimera states has previously been observed in systems of coupled hair bundles^42^. These chimera states are defined as systems in which a subset of the coupled oscillators show mutual synchronization, while the rest oscillate incoherently^43^. As hair cells inherently possess heterogeneity in their size, structure, and time scales of ion-channel dynamics, systems of coupled hair bundles are unlikely to exhibit full synchronization but are rather poised to support partial synchronization. As chimera states can lead to high sensitivity of detection^42,44^, we investigated the effect of efferent activation on this phenomenon. The rearrangement of the CC matrix allows one to visualize the clusters of synchronized hair cells and determine if efferent activity affects the clustering pattern. Figure 9 shows the particular cases for a 50 and a 150 µA step stimulus. For step stimuli, no variation was observed in the cluster distribution, despite an overall decrease in CC values. Hence, regardless of the reduction of inter-cell synchronization, the chimera states persisted under efferent stimulation. The observed behavior was consistent across all experiments, including those with high amplitudes of efferent activation (Fig 9B).

## Discussion

We have shown how systems of coupled hair bundles are impacted by activation of efferent neurons in the bullfrog sacculus. We were able to couple hair cells with artificial membranes and monitor their active, innate motility optically, without physical disturbance in the epithelium, thus maintaining cells’ integrity. We found that efferent activation had a significant impact on the coupled hair bundles, with the effects varying according to the stimulus waveform applied.

While hair cells as individual active detectors have been the focus of previous studies, in nature, sensory epithelia typically exhibit inter-cell coupling to enhance the detection of weak signals^13,45^. One can use a hybrid *in vitro* preparation to mimic the coupling observed *in vivo*, not only in the bullfrog sacculus, but across various species. This approach hence introduces array size as parameter that can be controlled by experimental conditions, which is not provided by physiological coupling. For this study, we chose to constrain our system size, selected to estimate the number of hair cells innervated by an efferent neuron in the sacculus^28^, while leaving room for control cells in the immediate vicinity of the coupled system.

Pulse train electrical stimulus with a square wave creates specific time windows during which efferents are depolarized. Note that the firing rate of efferents is not explicitly controlled, but rather a periodic modulation of spiking probability is introduced. Notably, these experiments showed entrainment of hair bundle motility to the pulse train stimulating the efferent neurons. While the exact mechanism of how efferents affect bundle motility has not yet been fully established, prior studies have explored various components of this process. As discussed above, the primary effect of this neural feedback is hyperpolarization of the hair cell somae. Electrophysiological manipulation of the membrane potential in turn was shown to induce movements of the hair bundle of tens of nanometers and more. The effect was shown to be calcium dependent, indicating that changes in the voltage affect the bundle mechanics by controlling the influx of calcium through the transduction channels. The current study shows that efferent feedback control of bundle motility can exert time-dependent modulation, yielding phase-locking to the efferent signals in the 5-100 Hz frequency range. When viewing the efferent system as a way of controlling the dynamics state of this sensory system, we note that these findings are consistent with theoretical models of the hair cell. In these models, the response of the hair cell has been described using nonlinear dynamics theory, and specifically equations that capture the Hopf bifurcation. In this theoretical framework, a control parameter determines the dynamic state of the cell, poising it in the quiescent or oscillatory regime. One study has shown that introducing dynamic modulation of a control parameter (parametric forcing) likewise elicits a phase-locked response of the hair bundle^46^. Since efference may constitute the biological counterpart to the control parameter in theoretical models, the entrainment of hair bundle movement observed in these experiments is consistent with theoretical models of parametric forcing.

Our experiments furthermore allowed us to compare the ability of coupled and uncoupled cells to entrain to the efferent signals. Interestingly, as the frequency of the stimulus increased, there was a markedly stronger capability of the coupled system to entrain to the higher frequencies (up to 100 Hz, the maximum frequency in our experiment), compared to the free-standing, control cells. Free-standing saccular hair bundles naturally oscillate at a broad range of frequencies, centered around roughly 30 Hz^47^. At the 40 Hz signal, which approximates the average frequency of natural spontaneous oscillations in the sacculus, control cells reach their maximum frequency increase due to phase-locking. Control hair bundles exhibit phase-locking behavior reflecting only individual response, as there is no mechanical interaction among neighboring hair cells that would influence their active dynamics. We speculate that, as hair cells within the coupled system are constantly adapting to their adjacent neighbors, the collective response may have a heterodyning effect, allowing them to follow the electrical input at higher pulse frequencies. Consistent with both traits, the number of coupled cells that entrained to an applied efferent stimulus peaked at a 20-40 Hz pulse signal and decreased progressively away from the cells’ natural frequency (Fig. 8E). Hence, while they maintain phase-locking over a broader range, hair bundles are more susceptible to efferent entrainment when the signal is close to their inherent frequency.

Intermittent mode-locking was found only in uncoupled, control cells. The top trace in Fig. 5 exhibits a 2:1 phase-locking ratio, while coupled hair bundles oscillated 1:1 with the stimulus frequency. This phenomenon of multi-mode locking has been previously observed with mechanical stimulation and proposed as a potential mechanism for encoding detection of frequencies beyond the natural oscillatory spectrum of a single cell^48^. With electrical stimulation of the efferent neurons, the multi-mode locking was suppressed for coupled hair bundles, and the collective response fully entrained to the pulsed efferent signal. The profile of the evoked bundle motion recalls a superposition of the applied signal and the pre-stimulus oscillations.

Changes in amplitude and the opening probability remained similar for both coupled and control cells. Both parameters presented decreasing trends, with the open probability showing a stronger reduction for coupled cells. This shift led to spike-shaped oscillations, which has been shown to correlate with a weaker influx of ions into the stereocilia^39^. Reduction of opening probability in the coupled system could contribute to a more effective dampening of sensitivity by means of efferent activation^49,50^.

The application of a continuous step to the efferent nerve bundle does not introduce entrainment of the hair bundles. It does, however, strongly modulate the oscillation characteristics of individual bundles, thus potentially affecting inter-cell synchronization. Coupled bundles lost correlation with increasing stimulus intensity, but did not cross the coupling threshold, thus remaining synchronized. Furthermore, the effects on oscillation profiles of the coupled cells were weaker compared to the control cells. The increase in the oscillation frequency and the decrease of the amplitude of motion showed a change in the slope, for higher currents (above 100 µA). Similar effects were observed for both control and coupled hair bundles, although a higher frequency increase and stronger amplitude reduction were observed for control hair bundles. Comparable trends were also observed for the open probability parameter.

It has been proposed that hair bundles use chaotic dynamics in order to achieve high temporal acuity and sensitivity to small perturbations, with optimal sensitivity occurring in the weakly chaotic regime^37,41^. Quantifying the level of chaos using experimental measurements can be achieved by estimating the Kolmogorov entropy, where higher values indicate more chaotic, unpredictable behavior. We therefore employed the KE as an additional metric for characterizing the effects of efferent activation on hair-bundle dynamics. The KE of individual, uncoupled hair bundles increased in a non-monotonic manner with increasing constant-current stimulus levels. This type of stimulus likely increased the spontaneous spiking rates of the efferent neurons, without producing the entraining effect that arises from the pulse-train stimulus. As expected, this increase in KE was accompanied by a more irregular spontaneous oscillation profile and a broader power spectrum.

Upon mechanically coupling groups of hair bundles in the absence of stimulus, the KE increased, likely due to the increase in the number of degrees of freedom characterizing the coupled system. However, with efferent activation, the coupled system displayed a narrower range of KE values, and exhibited a monotonic increase in KE with increasing constant-current stimulus levels. We therefore speculate that *in vivo* efferent activity may be responsible for modulating the level of chaos in hair-cell dynamics, thereby adjusting the sensitivity, in order to detect weak signals, or to attenuate strong signals and protect the system from damage.

Partial synchronization, in the form of frequency-clustering states and chimera states, has also been proposed to be an important element of signal detection by auditory and vestibular systems^42,44^. Surprisingly, we found that constant-current efferent stimulus preserves these synchronization patterns while drastically altering the oscillation profiles and oscillation frequencies of the hair bundles (Fig. 9). Future work entails exploring how these synchronization patterns are influenced by a number of different efferent stimulus types, including stochastic spiking, chaotic spiking, and high-frequency bursts of spikes.

The inhibitory nature of the efferent system has direct implications in active hair bundle motility, and those responses can be used to probe its machinery. Efferents were shown to play a role in shaping not only the amplitude and frequency of spontaneous oscillations, but also entrainment and inter-cell synchronization. First, the amplitude of oscillation was smaller, indicating decreased hair bundle activity. Secondly, the data indicate a reduction in the mean opening probability of transduction channels, consistent with a decreased mechanical sensitivity of the hair cell. Finally, a decrease of synchronization between coupled hair bundles was consistently observed, which may provide an additional mechanism by which efferent activation reduces the sensitivity of the coupled system. Future work will explore the application of mechanical stimuli to the artificial membrane while applying efferent activation, to study its effects on the sensitivity. Coupled systems present a highly useful and relevant experimental scenario, as little is currently known of the effects of efferent activation on the collective active motility of auditory and vestibular sensory cells.

## Supplementary Information

Supplementary Information.

## Data Availability

Data sets generated during the current study are available from the corresponding author on reasonable request.
