# Supplementary material for: Efferent control of hair cells mechanically coupled by artificial membranes
Source: Sci Rep. 2025 Dec 13;16:1594. doi: 10.1038/s41598-025-31059-1 (PMC12800259; doi:10.1038/s41598-025-31059-1)
Supplement: Supplementary file 1 — Supplementary Information. [file 41598_2025_31059_MOESM1_ESM.pdf]

Supplementary Information

Martín A. Toderi [1,*], Gabriela Muñoz-Hernandez[1], Justin Faber[1], and Dolores Bozovic[1,2]

[1]Department of Physics & Astronomy, University of California, Los Angeles, California, 90095, USA.
[2]California NanoSystems Institute, University of California, Los Angeles, California, 90095, USA.
[*]mtoderi@physics.ucla.edu

Title: Efferent control of hair cells mechanically coupled by artificial membranes

1 Consistency of efferent effects with varying size of the hair bundle array

We tested the influence of the system size on the efferent effect by finding the dependence of the amplitude, frequency, and correlation coefficient on the number of mechanically coupled hair bundles. In Fig. S1, we grouped 36 experiments from 2 sacculi, spanning systems of 3 to 13 coupled cells (cells that exhibited a correlation coefficient higher than 0.2 with the membrane motion). We observed no statistically significant trends, indicating that there was no dependence of the measured effects on the system size, consistent with previous experiments[1]. For clarity, we added linear fits to each stimulus intensity. We hence conclude that there is a consistent response over the array sizes explored in the study. In the figures presented in the main text, we therefore pool the results obtained from various sizes of the coupled systems.

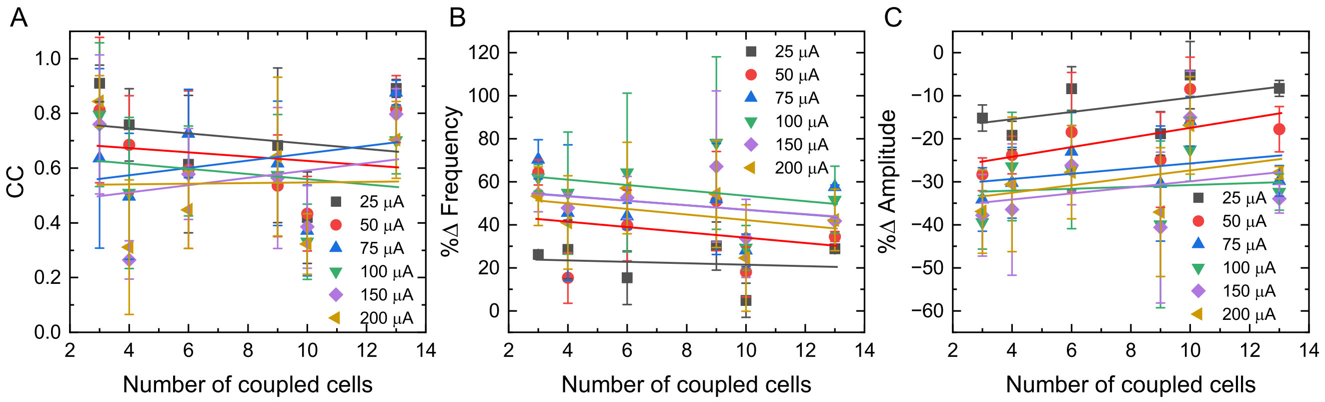

Figure S1. Relationship between the efferent effect and the size of the coupled system. Data is presented for electrical effererent stimulation in the form of continuous current steps, spanning 25 to 200 μA (for coupled cells satisfying a CC above 0.2 with the membrane motion). Straight lines are linear fits to the data, obtained for each stimulus intensity. Points and error bars correspond, respectively, to the means and standard deviations across experiments. (A) Correlation coefficient, (B) percent variation of frequency, and (C) percent variation of amplitude, versus the number of coupled hair bundles.

2 Control experiments verifying efferent activation

The importance of efferent synaptic activity in driving the observed effect was established through the introduction of strychnine into the basolateral compartment. Strychnine inhibits acetylcholine from interacting with nicotinic receptors (nAChRs) located on the hair cell soma, thereby nullifying the influence exerted by efferent synapses on the cell[2].

A stock solution of 10 mM concentration of strychnine hydrochloride ($C_{21}H_{22}N_2O_2 \cdot HCl$, Chem-Impex, CAS # 1421-86-9) was prepared in deionized water. Immediately before usage, a dilution of 2 μM was made in artificial perilymph and exchanged in the basal compartment of the sample chamber bathing the exposed nerve tissue, to induce blockage of the efferent effect. We tracked the HBs motion during two continuous step stimuli of 50 and 100 μA, with non-stimulated intervals of oscillation recorded before and after each current step. Figure S2A shows the initial motion traces for coupled hair bundles (red), the artificial membrane (black), and free-standing surrounding cells (blue). After 10 to 20 min of incubation, allowing sufficient perfusion of the strichnyne solution, the efferent effect was abolished (Fig. S2B). After careful but thorough rinsing of the preparation with artificial perilymph, the previously observed effects of efferent activation were recovered (Fig. S2C).

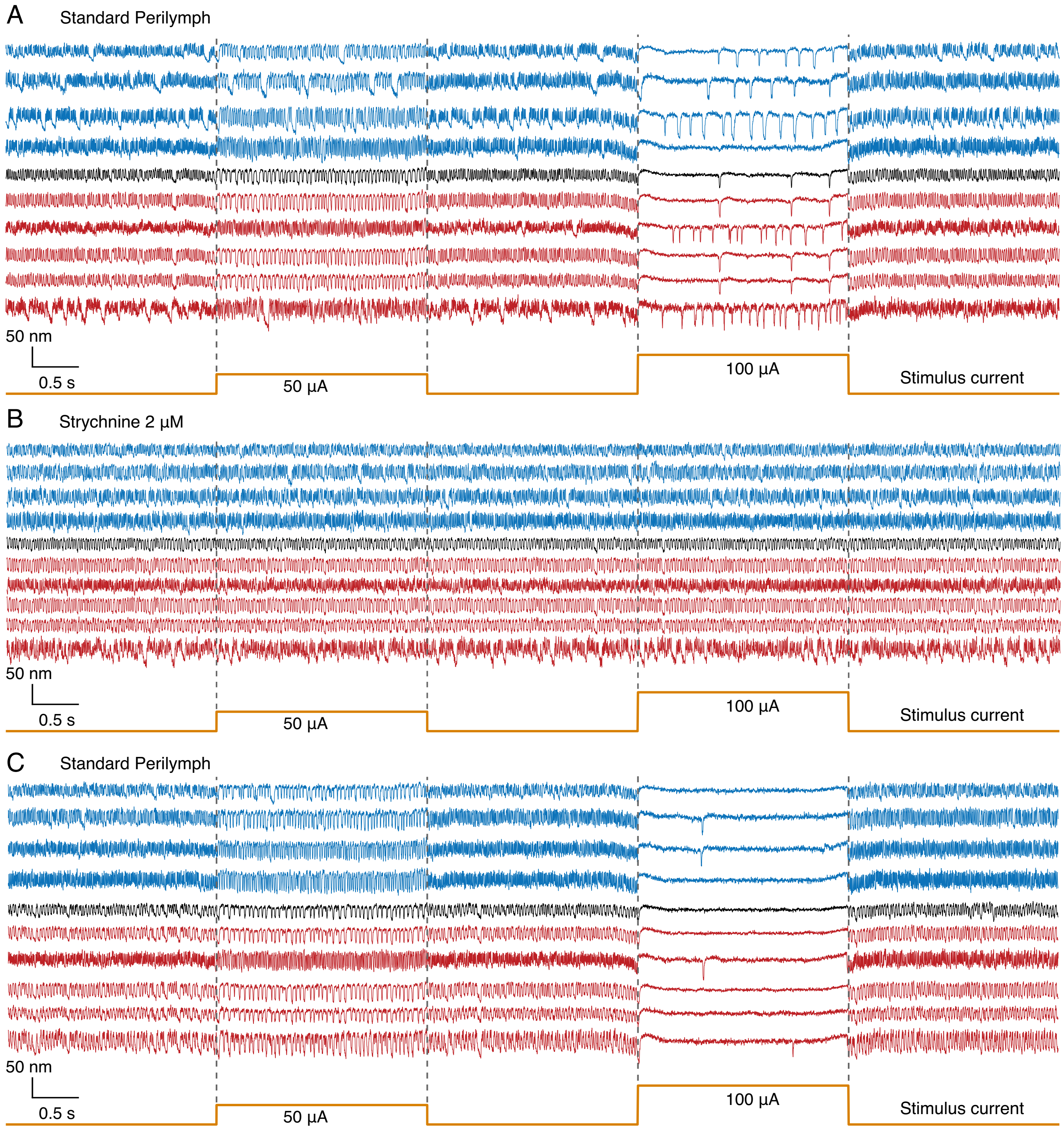

Figure S2. Control experiment for efferent stimulation under artificial coupling. Strychnine (2 μM) introduced into the artificial perilymph blocked effects of the electrical stimulus applied to the auditory nerve. The stimulus consisted of two steps (50 and 100 μA), each of 5 s in duration, with 5 s of non-stimulated time between them. 5 s of no stimulus were recorded before the first step and after the second. Vertical dashed lines mark the onsets and offsets of the stimulus. Cells under the artificial membrane are in red, the membrane motion in black, and surrounding free-standing cells in blue. (A) Shows the motion traces during application of efferent stimulation with standard Perilymph in the basal compartment. (B) Upon blockage of the receptors by adding Strychnine, the efferent stimulus exerts no visible effects on innate bundle motility. (C) Upon rinsing the Strychnine solution and restoring standard Perilymph, the effect is restored for both stimulus current-step intensities.

References

1. Faber, J., Li, H. & Bozovic, D. Synchronization and chaos in systems of coupled inner-ear hair cells. *Phys. Rev. Res.* **3**, 013266, DOI: 10.1103/PhysRevResearch.3.013266 (2021).

2. Guth, P., Dunn, A., Kronomer, K. & Norris, C. The cholinergic pharmacology of the frog saccule. *Hear. Res.* **75**, 225–232, DOI: https://doi.org/10.1016/0378-5955(94)90073-6 (1994).
